# Physical Activity and Associated Barriers/Facilitators in Older Adults Living in Northern Cyprus

**DOI:** 10.3390/healthcare14040527

**Published:** 2026-02-19

**Authors:** Beliz Belgen Kaygısız, Zehra Güçhan Topcu, Fahriye Çoban, Havva Gözgen, Necati Özler, Nurcihan Altun, Emine Ahsen Şenol, Aydın Meriç, Alavuddin Kurbonboyev

**Affiliations:** 1 Department of Physiotherapy and Rehabilitation, Faculty of Health Sciences, European University of Lefke, Lefke, Northern Cyprus, TR-10 Mersin, Turkey; fcoban@eul.edu.tr (F.Ç.); nozler@eul.edu.tr (N.Ö.); americ@eul.edu.tr (A.M.);; 2 Department of Physiotherapy and Rehabilitation, Faculty of Health Sciences, Eastern Mediterranean University, Famagusta, Northern Cyprus, TR-10 Mersin, Turkey; zehra.guchan@emu.edu.tr; 3 Department of Physiotherapy and Rehabilitation, Faculty of Health Sciences, Cyprus International University, Nicosia, Cyprus, TR-10 Mersin, Turkey; hgozgen@ciu.edu.tr; 4 Physioworld Fizyoterapi ve Sağlıklı Yaşam Merkezi, İskele, Northern Cyprus, TR-10 Mersin, Turkey; physioworld.cyprus@gmail.com; 5 Department of Physiotherapy and Rehabilitation, Faculty of Health Sciences, University of Kyrenia, Kyrenia, Northern Cyprus, TR-10 Mersin, Turkey; emineahsen.senol@kyrenia.edu.tr

**Keywords:** older adults, physical activity, exercise, sports

## Abstract

Background/Objectives: Keeping physical activity (PA) at an optimal level is important for protecting independence and keeping quality of life at the highest level while adopting healthy aging. This study aimed to estimate the PA levels of the older population living in Northern Cyprus and to examine the relationship between barrier and facilitator factors and PA levels. This is a cross-sectional population-based study. Methods: Detailed sociodemographic information was recorded and the PA level of the participants was evaluated using the Physical Activity Scale for the Elderly (PASE). Facilitators and barriers to participation in PA were assessed by questionnaire. Results: A total of 387 older individuals (224 women and 163 men; mean age: 74.3 ± 3.12) were grouped as youngest-old (68.31 ± 3.05) and old (80.29 ± 3.21). PA frequency and PASE scores were low in the overall study population and they were higher in the youngest-old group (*p* < 0.001). Conclusions: Low PA levels were estimated in older adults living in Northern Cyprus and they were significantly higher in the youngest-old group compared with the old group. There is a relationship between PA level and barriers such as fear of falling, fear of injury, comorbidities, shortness of breath, heart palpitations, incontinence, not knowing the benefits of PA, financial problems, not driving, and doing PA alone. Facilitators such as weight loss; living in a walkable area; friendship with groups; and being active, energetic, happy, and safe are related to PA level. This study highlights the importance of increasing PA levels by increasing awareness of the importance of PA in the older adult population, removing the barriers and using facilitators.

## 1. Introduction

Active and healthy aging is characterized as an optimization process of health, participation and safety opportunities, aimed at promoting quality of life during the aging process, and can be achieved through the performance of physical activities [1]. It has been strongly proven that individuals over 60 years of age who adopt an active lifestyle are associated with healthy aging [2]. Studies have proven that exercise and PA prevent falls and reduce pain, sarcopenia, osteoporosis, and even cognitive impairment [3,4,5,6]. In a longitudinal cohort study conducted between 1990 and 2008, where individuals aged 70–88 were included, it was revealed that the 8-year mortality rate was found to be almost 50% lower in physically active individuals [7].

On the other hand, physical inactivity is shown to be responsible for 9% of all deaths worldwide [8]. Physical inactivity is a major modifiable risk factor for non-communicable diseases and mental health conditions, including stroke, hypertension, type 2 diabetes, coronary heart disease, several types of cancers, dementia, depression, and all-cause mortality—in particular, deaths due to cardiovascular diseases [9].

A recent study by Zhou et al. revealed that a lack of regular PA has various negative effects on overall health, including a decrease in physical function, mobility and independence [10]. All these negative consequences of insufficient PA increase the health needs of the older population, which in turn results in an increase in the expenditures of the health system.

The strong association between PA and non-communicable diseases has prompted numerous countries to adopt strategies aimed at raising awareness of PA’s advantages. As part of the nine global targets to enhance the prevention and management of non-communicable diseases, World Health Organization (WHO) member states committed to achieving a 10% reduction in the prevalence of insufficient PA by 2025 [11]. Tracking current levels and trends of inadequate PA is crucial not only for monitoring progress toward this global target but also for identifying high-risk groups, evaluating the impact of policies, and shaping future policy and program development [12]. Identifying country-specific PA barriers and facilitators for older adults and better understanding their experience, views, and attitudes toward participating in PA can help us build strong evidence of the increasing PA participation of older adults in their own environment. At the same time, identifying motivators in achieving regular PA of older individuals will give us the option to increase PA participation. To our knowledge, there is no population-based study about PA level and related factors of older adults living in Northern Cyprus. So the purpose of our study is to estimate PA levels of older adults living in Northern Cyprus according to age and region and to examine the relationship between the barrier and facilitator factors and PA level. We hypothesized that PA level is associated with barrier and facilitator factors in the geriatric population.

## 2. Materials and Methods

### 2.1. Study Design

The study was a cross-sectional survey study. Individuals aged 65 years and older living in Northern Cyprus were invited to participate in the study. Individuals who agreed to participate in the study and met the inclusion criteria were evaluated. Before the study, all individuals were informed by the researcher about the study protocol. All participants signed a consent form before participation, and the rights of the participants were protected. This study was approved by the Ethical Research Committee of the European University of Lefke (No: UEK/52/02/07/1920/02).

### 2.2. Subjects

A total of 485 older adults were contacted through the municipalities of local regions by the researchers and invited to participate. Inclusion criteria were determined as being 65 years and older, living in Northern Cyprus, and living independently. Individuals who had undergone surgery in the past 6 months, individuals with symptomatic coronary artery disease, and individuals who had serious physical or mental health diagnoses were excluded. From those contacted, 38 participants refused to participate due to lack of time and motivation and 60 participants did not meet the inclusion criteria. As a result, 387 participants participated in this study.

### 2.3. Data Collection

Data collection started in August 2022 and was completed in October 2022 with the confirmed older adults in Northern Cyprus. It was collected from all six districts, including Famagusta, Trikomo, Kyrenia, Nicosia, Morphou and Lefka, by five interviewers. Six of the authors, who were physiotherapists with at least 2 years’ experience, were trained before the study to administer the data collection process. Three eligible participants were assessed by all interviewers to test inter-rater reliability (Fleiss’ kappa (≥3 raters = 0.80)).

### 2.4. Measurements

#### 2.4.1. Physical and Sociodemographic Information

Physical and sociodemographic information, including age, gender, height, weight, body mass index (BMI), educational status, marital status, number of chronic diseases, amount of daily alcohol use, amount of daily smoking, number of children, number of grandchildren, and frequency of exercise/PA (day/week), was measured (where applicable) and recorded in a specially prepared form by researchers.

#### 2.4.2. Evaluation of Barriers and Facilitators

Facilitators and barriers were established by researchers depending on the previous studies in the literature [1,13]. The form included subsets of personal, environmental, physical, social, and psychosocial factors, and each item was marked as “Yes” or “No” (Table A1).

#### 2.4.3. Evaluation of Physical Activity Level

The PA level of the individuals participating in the study was evaluated with the Physical Activity Scale for the Elderly (PASE). It consists of three subheadings and 12 questions covering leisure time, housework activities, and work-related activities, where the overall PASE score ranges from 0 to 400 or more, and high scores show better PA levels [14]. The PASE has been found to be a valid and reliable tool for use with older adults [14]. The internal consistency of these items, as measured by Cronbach’s alpha, was 0.69, and construct validity was also established by correlating PASE scores with health status and physiologic measures [14]. Also, the test–retest reliability was 0.75 (95% CI = 0.69–0.80) [14]. PASE is a valid and reliable test available in the Turkish language which was used for the Turkish-speaking population in our study [15].

### 2.5. Statistical Analyses

All statistical analysis was completed using IBM SPSS Statistics version 25.0 (IBM Corp, Armonk, NY, USA). The normal distribution was confirmed by visual (histograms/probability graphs) and analytical methods (Kolmogorov–Smirnov/Shapiro–Wilk test). Descriptive data were presented as mean and SD, median and interquartile range, or number and frequency. Comparisons of quantitative variables between groups were performed using the independent sample t-test for normally distributed variables. Spearmen’s Rank Correlation analysis was used to examine relationships between PA level and barriers/facilitators. A one-way ANOVA was applied to compare PA levels between districts, followed by a post hoc analysis. The level of significance was determined as *p* < 0.05.

## 3. Results

A total of 387 participants, 224 women and 163 men, from all regions of Northern Cyprus were included in the study. The average age of participants was 80.29 ± 3.21, and the mean BMI was 29.40 ± 4.46. Regionally, 37 participants (66.95 ± 5.20) from Trikomo, 22 participants (74.00 ± 5.91) from Lefka, 30 participants (74.17 ± 7.14) from Morphou, 80 participants (71.33 ± 5.89) from Famagusta, 101 participants (73.78 ± 6.58) from Kyrenia, and 117 participants (71.65 ± 6.61) from Nicosia participated in our study.

Everyone over the age of 65 was considered old, but because there is a big difference between 65 and 85, social gerontologists have divided old age into three subcategories. It is divided into the subcategories: young-aged for 65–74 years old, middle-aged for 75–84 years old, and older adults over 85 years old [16]. In our study, we grouped the participants parallel to these subcategories as youngest-old (65–74) and old (75 and above). The distribution of sociodemographic characteristics of older adults according to region and age group is shown in Table 1. There is a significant difference between the youngest-old and old groups in terms of the history of disease, daily smoking, and the number of children and grandchildren (Table 1).

We evaluated the frequency of exercise or PA (times per day/week) and PA level with the PASE (score). The results of the distribution and comparison of PA, according to region and age group, are shown in Table 1. Low PA levels were estimated in older adults living in Northern Cyprus. All parameter results were found to be significantly higher in the youngest-old group compared to the old group.

In order to evaluate barriers to and facilitators of participation in PA, we used the form, which is shown in Appendix A (Table A1). The results of the answers of participants to this form are shown in Table 2. Most of the participants (77.4%) stated “fatigue” as a barrier to performing PA. The benefits of PA are recognized as a facilitator of PA (79.9%), and financial problems were not recognized as a barrier to performing PA (83.5%). In terms of environmental factors, 83.8% of the participants stated that “living in a walkable area” is a facilitator of PA. Living alone (20.9%) and social pressure (13.0%) as social factors were not effective in encouraging the performance of PA. As for psychosocial factors, “thinking they’re getting better” was a major facilitator for PA (80.3%).

The level of PA was examined to determine the difference in PA between rural and urban districts, and this comparison is shown in Table 3. Participants living in rural districts (Lefke, Morphou and Trikomo) had higher PA scores than participants living in urban districts (Nicosia, Famagusta and Kyrenia).

For the evaluation of the relationship between PA and barriers and facilitators, we only included the significant results in Table 4 since the table would be too long. Barriers, including balance problems, fear of falling, fear of injury, comorbidity, shortness of breath, heart palpitations, incontinence, not knowing the benefits of PA, financial situation, not driving, and doing physical activities alone, were found to be significantly associated with PA level. At the same time, facilitators such as weight loss, living in a walkable area, friendships in groups, being active, feeling energized, being happy, and feeling safe have a significant relationship with PA level.

## 4. Discussion

PA has been identified to have an important role in successful aging. Knowing the fact that member states of the WHO recently put a big effort into analyzing both country-specific and global PA characteristics, the present study investigated the estimated PA levels of older adults living in Northern Cyprus according to age and region and examined the relationship between barrier and facilitator factors and PA level. Since the literature consists of more data from developed countries [1,17,18,19] and the level of PA is proportional to the development level of the country, we think that providing support for the literature in this respect, by analyzing Northern Cyprus as a developing country, is valuable. Our study results reveal that the level of PA changes according to age and region and that the PA levels of the participants are related to certain barriers and facilitators, which supports our hypothesis.

The level of PA decreases as age progresses, and a lower level of PA may cause an increase in non-communicable diseases [20,21,22]. A study by Milanović et al. [21] revealed that the lower PA level of old people compared to the youngest-old is due to the reduction in muscle strength in both upper and lower limbs and changes in body fat percentage, flexibility, agility, and endurance. The literature supports the idea that the incorporation of PA into one’s lifestyle will reduce risk for chronic diseases and mortality while providing a means for primary disease prevention [23]. Our study results are parallel to the literature, as the PA level of the old group was found to be lower than that of the youngest-old group, and the older group also had more chronic diseases than the youngest-old group. According to the World Health Organization (WHO), individuals over the age of 65 are recommended to do at least 150–300 min of moderate-intensity aerobic activity per week, strengthening activities involving major muscles at least two days a week, and functional balancing and strengthening activities at least three days a week [24]. So, as a support for awareness, we provided PA recommendations to our study population at the end of our assessment session.

A recent study by Ahmadi et al. (2021) observed that those staying in urban areas had lower levels of PA compared to those who stayed in rural areas [25]. In our study, the PA level of those who stayed in Lefka and Morphou, which are rural areas, had the highest PA level, which is not a parallel result. We think that the people in these regions are more engaged in agriculture, which causes higher PA levels. The lowest level of PA was observed in urban areas such as Kyrenia and Famagusta. Although Nicosia is an urban area, we observed a high level of PA. We think that the PA level of this region is high because it is the capital of Northern Cyprus and there are many areas and more opportunities for PA. In a similar study conducted with children in Northern Cyprus, it was emphasized that park areas are important in increasing the level of PA. We estimate that we have obtained this result in the older adult population for similar reasons [26].

In a systematic review, Franco et al. (2015) listed barriers and facilitators affecting participation in PA [13]. However, the review focused more on barriers. We enhanced our survey by incorporating additional facilitators, recognizing that they are just as important as the barriers affecting participation in PA. We hypothesized that understanding these facilitators would aid in developing strategies to increase participation in PA.

Looking at the findings of barriers to and facilitators of physical factors in our study, pain and fatigue among the physical and health factors were two barriers that a high percentage of the participants stated. In a study done by Sun et al. (2020) [27], health conditions, including comorbid conditions, physical symptoms, and functional limitations, were important impediments for PA and intervention adherence [27]. In a study where barriers to and facilitators of adherence to group exercise in institutionalized older adults were analyzed, it was found that barriers like acute diseases, anxiety and agitation, depression, fear of injury, symptoms of muscle weakness, medication, relationship dynamics, disagreement within groups or unwillingness to continue, family expectations, and communication and socioeconomic status were reported [28,29]. Aronow et al. (2011) stated that older adults avoid exercise because the muscle pain after exercise disturbed older adults [30]. Explaining the changes that occur after exercise to the patients will help us to solve this problem. In a study conducted in South Asia, older adults stated that they often get tired while doing strengthening exercises and therefore take frequent breaks [31]. All these health-related barriers negatively affect the PA level of older adults. In our study, health-related barriers such as balance disorders, fear of falling, fear of injury, comorbidities, shortness of breath and incontinence were found to be associated with PA. As some of these health issues can be treated or prevented, it is necessary to facilitate the access of older adults to PA in terms of their health through good collaboration with a medical team.

In a systematic review analyzing barriers and facilitators associated with PA, the absence of social support was reported as a barrier to PA in high- and low-middle-income countries in the Middle East [32]. In our study, lack of support from others was determined as a barrier to PA, which is a parallel result to the literature. In the older adult population where social support is important, people should be motivated to participate in PA and necessary social support should be provided by group family and friend activities or civil society activities. Additionally, educating older individuals on how to cope with avoidable obstacles can enhance their willingness to be physically active, such as by teaching them methods to relieve fatigue after PA, such as stretching. In fact, the process of stretching itself is enjoyable, and static stretching exercises not only improve flexibility, but also carry low risk of injury.

Recognition of the benefits of PA played an important role as a facilitator from the personal factors, whereas the item “don’t like gyms” was the most commonly stated personal barrier. In the studies conducted, it has been stated that most of the participants are aware of the benefits of PA, but the biggest problem is initiating PA [13,19]. Among environmental factors, living in a walkable area was stated as a facilitator by the highest percentage of the participants, while stairs and lack of a safe walking area were other environmental barriers. Providing an accessible and safe environment all around the country should be a role of governments in order to increase awareness and facilitate PA. This support will be accompanied by a decrease in non-communicable diseases, which in turn will decrease health expenses.

In terms of social and psychosocial factors, facilitators were mentioned more than barriers. Friendship in groups as a social factor and feeling energized and being happy as psychosocial factors were popularly mentioned facilitators. In the study done by Sun et al. (2020) [27], it was shown that social support and goal setting are prominent themes that serve as a facilitator and motivator for PA adherence. Also, enjoyment of the social aspects of PA and encouragement and companionship from others are factors that facilitate adherence to PA intervention [27]. In the study by Coelho et al. (2017), it was found that the most important factor facilitating the participation of individuals over the age of 60 in PA is well-being [1]. Also, it has been reported that PA is of great importance in reducing unwanted psychological factors such as anxiety and depression [2,33] and these factors are highly related. So there is a parallel benefit where increasing PA will result in less psychological distress and feeling better is a facilitator of PA. Apart from physical factors, social and psychological factors should be considered when increasing PA levels of older adult populations.

## 5. Limitations

Our study has several limitations which should be taken into account prior to the interpretation of our results. First of all, potential limitations arise from the fact that this study is questionnaire-based (barriers and facilitators) and there is a potential for information bias to occur. Second, we employed a cross-sectional design which cannot assume causality. Additionally, we did not included the participants with physical or mental health diagnoses, so the PA levels of this sample may not be representative of all older adults in the population. Furthermore, regarding the PA measure, the PASE results enable us to find out how often the respondents engaged in selected PA but do not provide information about the type of activities in which individuals were involved other than what is stated in the tool. Investigating these factors for various types of PA could offer a clearer understanding of which activities are more preferable and/or most suitable for older adults.

## 6. Conclusions

In our study, PA levels of older adults living in Northern Cyprus were estimated. Our results show that the PA levels of older adults are low and decrease by age and that those living in rural regions have higher PA levels. This study also identified many different barriers and facilitators at the individual level and community level. It was revealed that health-related barriers have a higher correlation with decreased PA levels, which highlights the importance of collaboration with a medical team. Increasing awareness of the importance of optimal PA levels and decreasing risk factors for a sedentary lifestyle are very important. Both individual-based and community-based barriers need to be modified with the necessary multidisciplinary approaches and facilitators should be promoted. The literature is limited about the levels and determinants of PA in middle- and low-income countries, so we suggest conducting more PA studies for these countries. Also, further research is needed to identify potential policy actions that may increase the adoption of physically active lifestyles and implement healthy aging policies in order to increase the quality of life of older adults.

## Figures and Tables

**Table 1:** Distribution and comparison of sociodemographic characteristics, physical activity level and frequency of physical activity of elderly participants according to region and age group.

Parameters	Trikomo (n = 37)			Lefke (n = 22)			Morphou (n = 30)			Famagusta (n = 80)		
	Youngest-Old (65–74 Year)	Old (≥75 Year)	*t*-Test	Youngest-Old (65–74 Year)	Old (≥75 Year)	*t*-Test	Youngest-Old (65–74 Year)	Old (≥75 Year)	*t*-Test	Youngest-Old (65–74 Year)	Old (≥75 Year)	*t*-Test
	$\overset{-}{x}$ ± SD	$\overset{-}{x}$ ± SD	*p*	$\overset{-}{x}$ ± SD	$\overset{-}{x}$ ± SD	*p*	$\overset{-}{x}$ ± SD	$\overset{-}{x}$ ± SD	*p*	$\overset{-}{x}$ ± SD	$\overset{-}{x}$ ± SD	*p*
Age	68.23 ± 3.35	78.83 ± 3.76	0.00 **	68.55 ± 3.50	78.29 ± 3.17	0.00 **	68.59 ± 3.20	81.46 ± 2.87	0.00 **	67.93 ± 2.87	79.08 ± 2.78	0.00 **
BMI	31.41 ± 4.82	30.38 ± 4.00	0.63	29.20 ± 5.47	29.55 ± 4.93	0.87	26.38 ± 4.34	28.16 ± 3.81	0.51	28.04 ± 4.01	29.33 ± 6.37	0.24
Chronic disease (n)	1.52 ± 0.96	1.83 ± 0.75	0.45	1.82 ± 1.33	1.50 ± 0.85	0.48	0.76 ± 0.66	1.54 ± 0.78	0.01 *	1.49 ± 0.93	1.60 ± 1.19	0.65
Daily alcohol use (IU/day)	0.03 ± 0.18	0.00 ± 00	0.00 **	0.18 ± 0.60	0.50 ± 1.87	0.60	0.12 ± 0.49	0.00 ± 00	0.00 **	0.18 ± 0.57	0.04 ± 0.20	0.25
Daily smoking (pcs/day)	1.61 ± 5.23	1.67 ± 4.08	0.98	3.64 ± 8.09	2.86 ± 6.11	0.79	3.24 ± 6.83	0.77 ± 2.77	0.23	2.72 ± 5.42	2.52 ± 5.21	0.87
Children (n)	4.06 ± 1.88	4.83 ± 1.72	0.36	1.64 ± 1.03	2.29 ± 1.90	0.32	2.06 ± 0.66	4.15 ± 1.52	0.00 **	2.51 ± 1.74	2.96 ± 1.90	0.29
Grandchildren (n)	5.77 ± 3.19	8.17 ± 4.62	0.13	2.09 ± 1.92	3.64 ± 3.15	0.17	2.35 ± 2.18	9.38 ± 6.28	0.00 **	3.75 ± 3.73	4.88 ± 4.20	0.23
Frequency of exercise/PA (day/week)	2.48 ± 2.80	1.67 ± 2.88	0.52	1.00 ± 1.41	0.29 ± 0.83	0.13	3.06 ± 2.59	1.08 ± 2.22	0.04 *	2.63 ± 2.93	1.92 ± 2.93	0.31
PASE (score)	79.46 ± 42.39	35.54 ± 31.6	0.02 *	156.2 ± 59.23	85.36 ± 41.96	0.00 **	128.4 ± 63.56	65.65 ± 31.96	0.02 *	54.26 ± 51.15	37.24 ± 27.91	0.12
**Parameters**	**Kyrenia (n = 101)**			**Nicosia (n = 117)**			**Total (n = 387)**					
	**Youngest-Old** **(65–74 Year)**	**Old** **(≥75 Year)**	***t*** **-Test**	**Youngest-Old** **(65–74 Year)**	**Old** **(≥75 Year)**	***t*** **-Test**	**Youngest-Old** **(65–74 Year)**	**Old** **(≥75 Year)**	***t*** **-Test**			
	**$\overset{-}{x}$ ± SD**	**$\overset{-}{x}$ ± SD**	***p***	**$\overset{-}{x}$ ± SD**	**$\overset{-}{x}$ ± SD**	***p***	**$\overset{-}{x}$ ± SD**	**$\overset{-}{x}$ ± SD**	***p***			
Age	69.06 ± 2.88	80.98 ± 3.24	0.00 **	67.98 ± 3.07	80.94 ± 3.01	0.00 **	68.31 ± 3.05	80.29 ± 3.21	0.00 **			
BMI	29.82 ± 4.59	30.34 ± 3.78	0.55	28.17 ± 4.72	28.57 ± 3.63	0.65	28.83 ± 4.71	29.40 ± 4.46	0.25			
Chronic disease (n)	2.13 ± 1.05	2.67 ± 1.22	0.02 *	1.58 ± 0.93	2.03 ± 0.91	0.02 *	1.61 ± 1.02	2.04 ± 1.13	0.00 **			
Daily alcohol use (IU/day)	0.52 ± 1.43	0.45 ± 1.35	0.82	0.40 ± 1.25	0.06 ± 0.23	0.11	0.31 ± 1.06	0.21 ± 0.98	0.37			
Daily smoking (pcs/day)	5.78 ± 10.32	2.98 ± 7.89	0.14	6.81 ± 10.84	1.67 ± 6.09	0.01 *	4.76 ± 9.07	2.26 ± 6.23	0.00 **			
Children (n)	2.72 ± 1.23	3.71 ± 1.33	0.00 **	2.16 ± 0.86	2.92 ± 0.91	0.00 **	2.56 ± 1.43	3.31 ± 1.57	0.00 **			
Grandchildren (n)	4.39 ± 2.88	6.43 ± 2.57	0.00 **	2.57 ± 2.22	4.97 ± 2.50	0.00 **	3.56 ± 3.05	5.83 ± 3.80	0.00 **			
Frequency of exercise/PA (day/week)	1.92 ± 2.38	0.81 ± 1.88	0.01 *	1.56 ± 2.36	1.33 ± 2.45	0.63	2.05 ± 2.56	1.16 ± 2.28	0.00 **			
PASE (score)	89.68 ± 36.36	40.90 ± 42.91	0.00 **	128.1 ± 58.01	82.98 ± 60.79	0.00 **	99.1 ± 59.25	58.07 ± 48.93	0.00 **			

$\overset{-}{x}$, Mean; SD, Standard Deviation; BMI, Body Mass Index; IU, International Unit (10 gr); PASE, Physical Activity Scale for the Elderly, *, correlation is significant at the 0.05 level; **, correlation is significant at the 0.01 level.

**Table 2:** Frequency of barrier and facilitator factors of participants for physical activity.

Barriers	Yes	No	Facilitators	Yes	No
	*n* (%)	*n* (%)		*n* (%)	*n* (%)
Physical and Health Factors					
Pain	307 (75.4)	100 (24.6)	Pain	148 (36.4)	259 (63.6)
Fatigue	315 (77.4)	92 (22.6)	Potential prevention of health problems	284 (69.8)	123 (30.2)
Balance problems	216 (53.1)	191 (46.9)	Maintaining balance and strength	300 (73.7)	107 (26.3)
Fear of injury	204 (50.1)	203 (49.9)	Weight loss	285 (70.0)	122 (30.0)
Comorbidity	168 (41.3)	239 (58.7)			
Shortness of breath	129 (31.7)	278 (68.3)			
Heart palpitations	116 (28.5)	291 (71.5)			
Incontinence	125 (30.7)	282 (69.3)			
Personal Factors					
Lack of motivation	245 (60.2)	162 (39.8)	Not enjoying physical activity	274 (67.3)	133 (32.7)
Don’t like gyms	252 (61.9)	155 (38.1)	Recognizing the benefits of physical activity	325 (79.9)	82 (20.1)
Dislike of group PA	216 (53.1)	191 (46.9)	Sense of self-confidence	248 (60.9)	159 (39.1)
Not being used to PA	214 (52.6)	193 (474)	Support of others	185 (45.5)	222 (54.5)
Don’t know the benefits of PA	134 (32.9)	273 (67.1)			
Too hard (difficult)	99 (24.3)	308 (78.7)			
Financial problems	67 (16.5)	340 (83.5)			
Driving a vehicle	111 (27.3)	296 (72.7)			
Environmental Factors					
Lack of places for PA in the area where lives	194 (47.7)	213 (52.3)	Living in a walkable area	341 (83.8)	66 (16.2)
Lack of sports center	180 (44.2)	227 (55.8)	PA locations are close	257 (63.1)	150 (36.9)
Lack of access to the PA site	174 (42.8)	233 (57.2)	Convenient PA options at home	224 (55.0)	183 (45.0)
Stairs	281 (69.0)	126 (31.0)	Pleasant weather	275 (67.6)	132 (32.4)
Parking problem	215 (52.8)	192 (47.2)	Lack of alternatives in bad weather	159 (39.1)	248 (60.9)
Ground problem	275 (67.6)	132 (32.4)	Lack of places to rest outside	166 (40.8)	241 (59.2)
Inappropriate PA locations	272 (66.8)	135 (33.2)	PA locations are safe	205 (50.4)	202 (49.6)
No sidewalks	273 (67.1)	134 (32.9)			
Lack of safe walking area	279 (68.6)	128 (31.4)			
Traffic	264 (64.9)	143 (35.1)			
Social Factors					
Doing PA alone	165 (40.5)	242 (59.5)	Friendship in groups	252 (69.8)	155 (38.1)
Not being active	180 (44.2)	227 (55.8)	Being active	235 (57.7)	172 (42.3)
Living alone	85 (20.9)	322 (79.1)	Social pressure	53 (13.0)	354 (87.0)
Psychosocial Factors					
Depression	126 (31.0)	281 (69.0)	Feeling energized	284 (69.8)	123 (30.2)
Mood change	175 (43.0)	232 (57.0)	Being happy	281 (69.0)	126 (31.0)
			Think you’re getting better	327 (80.3)	80 (197)
			Feeling safe	274 (67.3)	133 (32.7)

PA, Physical Activity.

**Table 3:** Comparison of physical activity levels between rural and urban districts.

Physical Activity Scale For The Elderly Total Score								
			Urban Districts					
			Nicosia (n = 117)		Famagusta (n = 80)		Kyrenia (n = 101)	
		$\overset{-}{x}$ ± SD	$\overset{-}{x}$ ± SD	*p*	$\overset{-}{x}$ ± SD	*p*	$\overset{-}{x}$ ± SD	*p*
Rural Districts	Trikomo (*n* = 37)	72.34 ± 43.59	115.30 ± 62.16	0.00 **	49.07 ± 45.85	0.24	70.35 ± 45.68	1.00
	Lefke (*n* = 22)	116.55 ± 60.87	115.30 ± 62.16	1.00	49.07 ± 45.85	0.00 **	70.35 ± 45.68	0.00 **
	Morphou (*n* = 30)	101.22 ± 60.49	115.30 ± 62.16	0.78	49.07 ± 45.85	0.00 **	70.35 ± 45.68	0.06

$\overset{-}{x}$, Mean; SD, Standard Deviation; **, significant at the 0.01 level (2-tailed). One-way ANOVA and post hoc Tukey test.

**Table 4:** Relationship between physical activity level of older population and barriers/facilitators.

Physical Activity Scale for the Elderly	r	*p*
Physical and Health Factors—Is a balance disorder a barrier to exercise/physical activity?	0.262	0.000 **
Physical and Health Factors—Is fear of falling a barrier to exercise/physical activity?	0.232	0.000 **
Physical and Health Factors—Is fear of injury a barrier to exercise/physical activity?	0.251	0.000 **
Physical and Health Factors—Is comorbidity a barrier to exercise/physical activity?	0.288	0.000 **
Physical and Health Factors—Is shortness of breath a barrier to exercise/physical activity?	0.251	0.000 **
Physical and Health Factors—Are heart palpitations a barrier to exercise/physical activity?	0.239	0.000 **
Physical and Health Factors—Is incontinence a barrier to exercise/physical activity?	0.278	0.000 **
Personal Factors—Is not knowing the benefits of physical activity a barrier to exercise/physical activity?	0.149	0.003 **
Personal Factors— Are financial problems a barrier to exercise/physical activity?	0.149	0.003 **
Personal Factors—Is not driving a barrier to exercise/physical activity?	0.166	0.001 **
Environmental Factors—Is the lack of pavements a barrier to exercise/physical activity?	−0.122	0.016 *
Environmental Factors—Is the lack of a safe walking area a barrier to exercise/physical activity?	−0.122	0.016 *
Social Factors—Is doing physical activity alone a barrier to exercise/physical activity?	−0.229	0.000 **
Physical and Health Factors—Is weight loss a facilitator for exercise/physical activity?	−0.189	0.000 **
Environmental Factors—Is living in a walkable area a facilitator for exercise/physical activity?	−0.190	0.000 **
Environmental Factors—Are pleasant weather conditions a facilitator for exercise/physical activity?	−0.103	0.042 *
Environmental Factors—Is the lack of alternatives in bad weather conditions a facilitator for exercise/physical activity?	−0.116	0.022 *
Social Factors—Is friendship in groups a facilitator for exercise/physical activity?	−0.165	0.001 **
Social Factors—Is being active a facilitator for exercise/physical activity?	−0.184	0.000 **
Psychosocial Factors—Is feeling energized a facilitator for exercise/physical activity?	−0.247	0.000 **
Psychosocial Factors—Is being happy a facilitator for exercise/physical activity?	−0.251	0.000 **
Psychosocial Factors—Is feeling safe a facilitator for exercise/physical activity?	−0.110	0.031 *

r, Spearman’s Rank Correlation. *, correlation is significant at the 0.05 level (2-tailed). **, correlation is significant at the 0.01 level (2-tailed).

## Data Availability

The data presented in this study are available on request from the corresponding author due to privacy and ethical restrictions.

## Abbreviations

The following abbreviations are used in this manuscript:

PA	Physical Activity
PASE	Physical Activity Scale for the Elderly
WHO	World Health Organization

## Appendix A

**Table A1:** Questionnaire of facilitators of and barriers to participation in physical activity.

Barriers	Yes	No	Facilitators	Yes	No
Physical and Health Factors					
Pain			Pain		
Fatigue			Potential prevention of health problems		
Balance problems			Maintaining balance and strength		
Fear of injury			Weight loss		
Comorbidity					
Shortness of breath					
Heart palpitations					
Incontinence					
Personal Factors					
Lack of motivation			Not enjoying physical activity		
Don’t like gyms			Recognizing the benefits of physical activity		
Dislike of group PA			Sense of self-confidence		
Not being used to PA			Support of others		
Don’t know the benefits of PA					
Too hard (difficult)					
Financial problems					
Driving a vehicle					
Environmental Factors					
Lack of places for PA in area where they live			Living in a walkable area		
Lack of sports center			PA locations are close		
Lack of access to the PA site			Convenient PA options at home		
Stairs			Pleasant weather		
Parking problem			Lack of alternatives in bad weather		
Ground problem			Lack of places to rest outside		
Inappropriate PA locations			PA locations are safe		
No sidewalks					
Lack of safe walking area					
Traffic					
Social Factors					
Doing PA alone			Friendship in groups		
Not being active			Being active		
Living alone			Social pressure		
Psychosocial Factors					
Depression			Feeling energized		
Mood change			Being happy		
			Think you’re getting better		
			Feeling safe		
